# Clinical analysis of pulmonary infection in hemodialysis patients

**DOI:** 10.3892/etm.2014.1646

**Published:** 2014-03-28

**Authors:** WEI REN, HUIXUAN PAN, PENG WANG, LEI LAN, WEI CHEN, YAN WANG, LIJUN NI, LI PENG

**Affiliations:** Department of Nephrology, Anhui Provincial Hospital Affiliated to Anhui Medical University, Hefei, Anhui 230001, P.R. China

**Keywords:** hemodialysis, pulmonary infection, sputum culture, drug sensitivity test

## Abstract

The present study aimed to investigate the pathogen distribution and drug resistance of lung infections in hemodialysis to guide clinical empirical pharmacy. The clinical data of 116 hemodialysis patients with pulmonary infection were analyzed. The majority of the 82 pathogens isolated from the sputa of patients were Gram-negative bacteria (accounting for 71.95%). The results of the drug sensitivity test suggested that Gram-negative bacilli had low resistance rates to piperacillin and tazobactam, imipenem and amikacin, while Gram-positive cocci had a low resistance rate to vancomycin. All resistance rates of the pathogens to other common antimicrobials were >50%. The pathogens resulting in lung infections in hemodialysis patients were mainly Gram-negative bacteria and were significantly resistant to various antibacterials. Results of the this study demonstrate that pathological examination should be performed as early as possible and effective antimicrobial agents should be chosen according to drug sensitivity test results.

## Introduction

With the development of diagnosis and treatment methods, and the improvement of renal replacement therapy technology, the detection and survival rates of patients with chronic kidney disease (CKD) have gradually increased. The number of patients undergoing blood dialysis has increased each year and the survival time for hemodialysis patients has been significantly prolonged. Therefore, the understanding of the long-term complications associated with hemodialysis has become increasingly important. The risk of infection for hemodialysis patients is markedly higher than that of the general population due to immune dysfunction following kidney failure. Lung infection is common and is the main factor affecting life quality and mortality (1–3). Therefore, analysis of the clinical characteristics, common pathogenic bacteria and antibiotic susceptibility of bacteria-associated lung infections in hemodialysis patients has become increasingly important for the treatment and prognosis of lung infections by clinicians. In the present study, the general conditions, bacterial epidemiology and drug sensitivity of 116 hemodialysis patients with pulmonary infection were retrospectively analyzed, providing a reference for clinical diagnosis and treatment.

## Subjects and methods

### Clinical data

In total, 116 hemodialysis patients with a lung infection were selected between January 2004 and December 2011 from the Anhui Provincial Hospital Affiliated to Anhui Medical Universtiy (Hefei, China). The patient group included 86 males and 30 females aged between 19 and 88 years, with a mean age of 61.4±16.7 years. This study was conducted in accordance with the Declaration of Helsinki and with approval from the ethics committee of the Anhui Provincial Hospital Affiliated to Anhui Medical University. Written informed consent was obtained from all participants.

### Diagnostic criteria

All patients met the V staging and diagnostic criteria of CKD according to the Kidney Disease Outcomes Quality Initiative guidelines (4) and all patients were being treated with hemodialysis. The diagnosis of pulmonary infection included the following symptoms: Fever, emerging respiratory symptoms or existing respiratory symptom aggravation, emerging wet and dry rales or existing rale increase in the lungs, leukocytosis increase or decrease or any of the aforementioned symptoms plus invasive changes in the lung parenchyma observed via X-ray. Other pulmonary diseases were excluded.

### Research methods

Gender, age, duration of hospitalization, clinical manifestations, laboratory parameters (including serum creatinine and blood urea nitrogen levels, and blood routine examination), chest X-ray or computed tomography (CT) results, deep sputum bacterial culture, drug sensitivity, antibiotic usage and additional indexes of patients, were collected.

Patients were required to gargle with salt water in the early morning prior to the extraction of deep sputum specimens. The specimen collection of sputa and secretions of the lower respiratory tract were performed under sterile conditions and samples were then stored in sterile boxes at room temperature and examined within 2 h. The required squamous cell of ≤10/LP (line pairs per mm, lp/mm) and polymorphonuclear leukocytes of >25/LP were selected as sputum specimens and analyzed at a low magnification under a microscope (Olympus, Co., Ltd., Tokyo, Japan). Bacterial culture and drug sensitivity tests were performed according to accepted methods using blood agar, Leventhal’s agar and McConkey’s medium, and incubated for 24 h at 37°C. Susceptibility to antibiotics was tested by an agar dilution method using standard criteria.

### Effect evaluation

Treatment effects were determined using the following guidelines: i) Clinical cure: Respiratory symptoms and signs disappearing, normal peripheral blood epistrophy, complete absorption of acute pulmonary inflammatory lesions in X-ray; ii) clinical improvement: >50% disappearance of respiratory symptoms and signs, absorbance of the majority of pulmonary acute inflammatory lesions and improved in the X-ray; iii) invalid therapy: Presence of symptoms and signs and no improvement in the X-ray examination, or the patient succumbed to their condition one week after drug therapy.

### Statistical analysis

SPSS software, version 17.0 (SPSS, Inc., Chicago, IL, USA) was used for analysis and measurement data are expressed as the mean ± standard deviation. Comparisons between groups were analyzed using the Student’s t-test. Measurement data are expressed as a percentage, the comparison of sample rates was performed by the χ^2^ test. P<0.05 was considered to indicate a statistically significant difference.

## Results

### General results

The majority of the 116 hemodialysis patients with pulmonary infection were aged between 60 and 74 years (n=47, accounting for 40.51%) followed by patients aged >75 years (n=28, accounting for 24.14%) (Table I). Chronic glomerulonephritis was the predominant kidney foundation disease (accounting for 42.24%), followed by hypertensive renal arteriosclerosis (accounting for 27.59%) and diabetic nephropathy (accounting for 24.14%) (Table II).

### Clinical performances

All patients showed symptoms of a cough and were producing sputa, 82 patients had a fever during the course of the disease and auscultation identified rales in the lungs of 78 cases. Chest X-ray and CT scanning showed new or progressive inflammatory lesions in the unilateral or bilateral lungs. Increased white blood cells (WBCs) were observed in 68 cases.

### Pathogen detection of deep cultured sputum

In the deep sputum culture of the 116 patients, 79 cases were positive for pathogenic bacteria and 17 species of bacteria were isolated from 82 pathogens. The classification of the detected pathogens is shown in Table III. The distribution of Gram-negative and Gram-positive bacteria are shown in Table IV.

### Drug susceptibility test results of cultured sputum

The drug resistance rates of the Gram-negative and Gram-positive bacteria to antimicrobials are shown in Table V. The results of the drug sensitivity test showed that the resistance rate of *Escherichia coli* to imipenem was 0%. *E. coli* resistance to piperacillin/tazobactam and amikacin and nitrofurantoin was also low (7.7, 15.4 and 30.8%, respectively), while the resistance rates to additional agents was >50%. The resistance rate of *Pseudomonas aeruginosa* to imipenem, piperacillin/tazobactam and amikacin was 0%. *P. aeruginosa* resistance to levofloxacin was also low (16.7%), while resistance to other commonly used antimicrobial drugs was high. The resistance rates of *Klebsiella pneumoniae* to piperacillin/tazobactam amikacin and imipenem were 0, 0 and 11.1%, respectively, and >65% to cephalosporins and β-lactam drugs. The resistance rate of *Staphylococcus aureus* to vancomycin was 0%. Resistance to nitrofurantoin, piperacillin/tazobactam and cotrimoxazole (sulfamethoxazole/trimethoprim) (0, 16.7 and 16.7%, respectively) was also low in *S. aureus*, while resistance to commonly used antibiotics was >50%. The resistance rate of fungi to itraconazole and fluconazole was 0%.

### Treatment and prognosis

Of the 116 patients, the total number of cured and effectively treated patients was 100 patients, and the total effective rate was 86.21% (8 cases were invalid and 8 patients succumbed to their condition, with a mortality rate of 6.90%). The mean age of mortality was 74.8±11.3 years, which included 6 males and 2 females. The primary disease included 2 cases with chronic glomerulonephritis, 2 cases with hypertensive renal arteriosclerosis and 4 cases with diabetic nephropathy. The constituent ratio of the primary disease in mortality cases is shown in Table VI.

## Discussion

As the survival time of hemodialysis patients has increased, the understanding of the long-term complications of end-stage renal disease has become increasingly important. Following cardiovascular and cerebrovascular complications, infection is the second most common cause of mortality for hemodialysis patients (5,6), particularly lung infection (7–9).

The main causes of pulmonary infection in hemodialysis patients are as follows (8,10): i) Increased duration of time spent in hospital increases contact with pathogens; ii) occurence of atherosclerosis, hypertension, malnutrition, chronic microinflammation, anemia and uremic toxins promotes heart failure and pulmonary edema (11–13); iii) increased respiratory secretions with dense sputa and increased alveolar fibrin exudation; and iv) airway mucosal barrier dysfunction, enabling pathogen invasion, which is conducive to the invasion and dissemination of respiratory tract bacteria; iv) immune dysfunction. Previous studies have identified that a reduced number of B lymphocytes and decreased ability to produce immunoglobulins can result in humoral immune abnormalities in uremia (14,15). In addition, a reduced number and the dysfunction of T lymphocytes (particularly T4 lymphocytes), as well as enhanced phagocytosis, chemotaxis and bactericidal capacity, which decreases monocytes and neutrophils, may result in a decline in cellular immune function in patients. Additionally, the vascular puncture or the implantation of a foreign object, such as a deep venous catheter during hemodialysis, increases the probability of infection. These factors may subsequently induce lung inflammation in hemodialysis patients and, once infection has occurred, the infection is difficult to control and progresses rapidly. Previous studies revealed that the mortality of patients with uremia and pneumonia was 14–16-fold higher than that of the general population (14,15).

The present study identified that lung infection in hemodialysis was largely caused by Gram-negative bacteria (accounting for 71.95%), followed by Gram-positive bacteria (accounting for 18.29%) and fungi (accounting for 9.76%), consistent with results of a previous study (8). The predominant Gram-negative bacilli was *E. coli* (15.85%), *P. aeruginosa* (14.63%), *K. pneumoniae* (10.98%) and *Haemophilus influenzae* (8.54%), while the main Gram-positive cocci was *S. aureus* (7.32%).

In previous years, due to the widespread use of antibiotics, pathogens are constantly changing and the number of drug-resistant pathogenic bacteria has augmented resulting in a significant increase in the resistance rates of numerous commonly used antibiotics (such as cephalosporins) (16–21). Consistent with this notion, the results of the drug sensitivity tests in the present study showed that the resistance rates of Gram-negative bacilli to piperacillin/tazobactam, imipenem and amikacin were low, and the resistance rates to the traditional β-lactam antibiotics and first and second generation cephalosporin antibiotics were >50%; therefore the utilization of these agents should be avoided. The resistance rate of Gram-positive cocci to vancomycin was low, as was resistance to nitrofurantoin, piperacillin/tazobactam and cotrimoxazole. The resistance rates to other commonly used antimicrobials were >50%. The resistance rates of various bacterium types to piperacillin/tazobactam were <30%. Itraconazole and fluconazole were universally effective against fungi.

The prognosis of hemodialysis patients with a lung infection was positive if treated promptly and effectively. In the present study, the clinical cure and improvement rates were as high as 86.21%. During this study, 8 patients succumbed to their condition due to invalid treatment and the mortality rate was 6.90%. Data analysis showed that the average age of mortality was 74.8±11.3 years and the mortality of hemodialysis patients with diabetic nephropathy as the primary disease was significantly higher than that of other diseases. Mortality was significantly increased when pulmonary infection occurred in elderly hemodialysis patients. In addition, mortality was associated with primary diseases where infection was difficult to control, with diabetic nephropathy as the primary disease most likely to result in mortality.

In conclusion, in the current study, the majority of pulmonary infections in hemodialysis patients were caused by Gram-negative bacteria. A variety of commonly used antimicrobial agents, including piperacillin/tazobactam, imipenem and amikacin, were found to be effective against Gram-negative bacilli, while vancomycin was effective against Gram-positive cocci. Nitrofurantoin, piperazine, piperacillin/tazobactam and cotrimoxazole were effective against the majority of Gram-positive cocci and itraconazole and fluconazole remained effective against fungi. These observations demonstrate that clinicians must determine the correct treatment agents according to the clinical drug susceptibility of pathogens.

The mortality of elderly patients with diabetic nephropathy, as well as hemodialysis patients with diabetic nephropathy, was significantly higher than that of the other patients; therefore, early diagnosis and early treatment should be administered to these patients to improve prognosis and reduce mortality.

In addition, more data are required to identify the clinical characteristics of pulmonary infection in patients receiving hemodialysis.

## Figures and Tables

**Table I:** Age distribution of 116 hemodialysis patients with pulmonary infections.

Age, years	n	Constitution ratio, %
<45	19	16.38
45–59	22	18.97
60–74	47	40.51
≥75	28	24.14

**Table II:** Basic diseases of 116 hemodialysis patients with pulmonary infections.

Basic diseases	n	Constitution ratio, %
Chronic glomerulonephritis	49	42.24
Hypertensive renal arteriosclerosis	32	27.59
Diabetic nephropathy	28	24.14
Other	7	6.03

**Table III:** Classifications of pathogens isolated from sputum culture.

Pathogens	n	Constitution ratio, %
Gram-negative bacteria	59	71.95
Gram-positive bacteria	15	18.29
Fungi	8	9.76

**Table IV:** Distributions of bacteria isolated from sputum.

Pathogens	n	Constitution ratio, %
Gram-negative bacteria		
*Escherichia coli*	13	15.85
*Pseudomonas aeruginosa*	12	14.63
*Klebsiella pneumoniae*	9	10.98
*Haemophilus influenzae*	7	8.54
*Acinetobacter baumannii*	5	6.10
*Enterobacter cloacae*	4	4.88
*Acinetobacter lwoffii*	4	4.88
*Proteus vulgaris*	2	2.44
*Enterobacter aerogenes*	1	1.22
*Citrobacter freundii*	1	1.22
*Aeromonas sobria*	1	1.22
Gram-positive bacteria		
*Staphylococcus aureus*	6	7.32
*Staphylococcus haemolyticus*	5	6.10
*Enterococcus faecalis*	2	2.44
*Streptococcus pneumoniae*	1	1.22
Aerobic bacillus	1	1.22

**Table V:** Resistance rates of major Gram-positive and Gram-negative bacteria to antimicrobial drugs (%).

	*Escherichia coli* (n=13)		*Pseudomonas aeruginosa* (n=12)		*Klebsiella pneumoniae* (n=9)		*Haemophilus influenzae* (n=7)		*Acinetobacter baumannii* (n=5)		*Staphylococcus aureus* (n=6)	
						
Antimicrobial drugs	n	Resistance rate	n	Resistance rate	n	Resistance rate	n	Resistance rate	n	Resistance rate	n	Resistance rate
Vancomycin	-	-	-	-	-	-	-	-	-	-	0	0.0
Piperacillin/tazobactam	1	7.7	0	0.0	0	0.0	2	28.6	1	20.0	1	16.7
Imipenem	0	0.0	0	0.0	1	11.1	1	14.3	1	20.0	4	66.7
Amikacin	2	15.4	0	0.0	0	0.0	2	28.6	0	0.0	3	50.0
Levofloxacin	12	92.3	2	16.7	3	33.3	4	57.1	3	60.0	3	50.0
Cotrimoxazole	11	84.6	12	100.0	4	44.4	3	42.9	2	40.0	1	16.7
Nitrofurantoin	4	30.8	12	100.0	6	66.7	6	85.7	5	100.0	0	0.0
Ceftriaxone	8	61.5	11	91.7	6	66.7	3	42.9	4	80.0	3	50.0
Cefazolin	10	76.9	12	100.0	7	77.8	7	100.0	5	100.0	5	83.3
Ampicillin	13	100.0	12	100.0	9	100.0	7	100.0	5	100.0	5	83.3

No drug sensitivity, (−).

**Table VI:** Constituent ratio of mortality cases.

Basic diseases	Cases, n	Mortality cases, n	Constituent ratio of mortality, %
Chronic glomerulonephritis	49	2	4.08^a^
Hypertensive renal arteriosclerosis	32	2	6.25^a^
Diabetic nephropathy	28	4	14.29

a P<0.01, vs. diabetic nephropathy patients.
